# Antimicrobial Potential of Aqueous Extract of Giant Sword Fern and Ultra-High-Performance Liquid Chromatography–High-Resolution Mass Spectrometry Analysis

**DOI:** 10.3390/molecules28166075

**Published:** 2023-08-15

**Authors:** Balu Alagar Venmathi Maran, Kishneth Palaniveloo, Thivyalaxmi Mahendran, Dinesh Kumar Chellappan, Jen Kit Tan, Yoong Soon Yong, Mohammad Tamrin Mohamad Lal, Elliecpearl Jasca Joning, Wei Sheng Chong, Olga Babich, Stanislav Sukhikh, Muhammad Dawood Shah

**Affiliations:** 1 Borneo Marine Research Institute, Universiti Malaysia Sabah, Jalan UMS, Kota Kinabalu 88450, Malaysia; bavmaran@ums.edu.my (B.A.V.M.); mdtamrin@ums.edu.my (M.T.M.L.); elliecpearlj.joning@gmail.com (E.J.J.); chong@ums.edu.my (W.S.C.); 2 Institute of Ocean and Earth Sciences, Advanced Studies Complex, Universiti Malaya, Kuala Lumpur 50603, Malaysia; kishneth@um.edu.my (K.P.); thivyalaxmi@gmail.com (T.M.); 3 Centre for Natural Products Research and Drug Discovery (CENAR), Level 3, Research Management & Innovation Complex, Universiti Malaya, Kuala Lumpur 50603, Malaysia; 4 Department of Life Sciences, School of Pharmacy, International Medical University, Kuala Lumpur 57000, Malaysia; dinesh_kumar@imu.edu.my; 5 Department of Biochemistry, Universiti Kebangsaan Malaysia Medical Centre, Jalan Yaacob Latif, Bandar Tun Razak, Cheras, Kuala Lumpur 56000, Malaysia; jenkittan@ukm.edu.my; 6 Faculty of Applied Sciences, UCSI University, Jalan Menara Gading, UCSI Heights, Cheras, Kuala Lumpur 56000, Malaysia; yongys@ucsiuniversity.edu.my; 7 Research and Education Center “Industrial Biotechnologies”, Immanuel Kant Baltic Federal University, A. Nevskogo Street 14, 236016 Kaliningrad, Russia; olich.43@mail.ru (O.B.); stas-asp@mail.ru (S.S.)

**Keywords:** antibacterial, antiparasitic, *Vibrio* spp., *Zeylanicobdella arugamensis*, metabolites, aquaculture, flavonoids, phenolics, giant sword fern, UHPLC-HRMS analysis

## Abstract

Vibriosis and parasitic leech infestations cause the death of various farmed fish, such as groupers, hybrid groupers, sea bass, etc., in Malaysia and other Southeast Asian countries. In the absence of natural control agents, aquaculture operators rely on toxic chemicals to control Vibrio infections and parasitic leeches, which can have a negative impact on the environment and health. In the present study, we investigated the antivibrio and antiparasitic activities of the aqueous extract of giant sword fern (GSF) (*Nephrolepis biserrata*, Nephrolepidaceae, locally known as “Paku Pedang”) against four *Vibrio* spp. and the parasitic leech *Zeylanicobdella arugamensis*, as well as its metabolic composition using the ultra-high-performance liquid chromatography–high-resolution mass spectrometry system (UHPLC-HRMS). The data show that the aqueous extract of GSF at a concentration of 100 mg/mL exhibits potent bactericidal activity against *V. parahaemolyticus* with a zone of inhibition of 19.5 mm. In addition, the extract showed dose-dependent activity against leeches, resulting in the complete killing of the parasitic leeches within a short period of 11–43 min when tested at concentrations ranging from 100 to 25 mg/mL. The UHPLC-HRMS analysis detected 118 metabolites in the aqueous extract of GSF. Flavonoids were the primary metabolites, followed by phenolic, aromatic, fatty acyl, terpenoid, vitamin and steroidal compounds. Notably, several of these metabolites possess antibacterial and antiparasitic properties, including cinnamaldehyde, cinnamic acid, apigenin, quercetin, cynaroside, luteolin, naringenin, wogonin, 6-gingerol, nicotinamide, abscisic acid, daidzein, salvianolic acid B, etc. Overall, our study shows the significant antibacterial and antiparasitic potential of the GSF aqueous extract, which demonstrates the presence of valuable secondary metabolites. Consequently, the aqueous extract is a promising natural alternative for the effective control of *Vibrio* infections and the treatment of parasitic leeches in aquaculture systems.

## 1. Introduction

**Figure 1:**
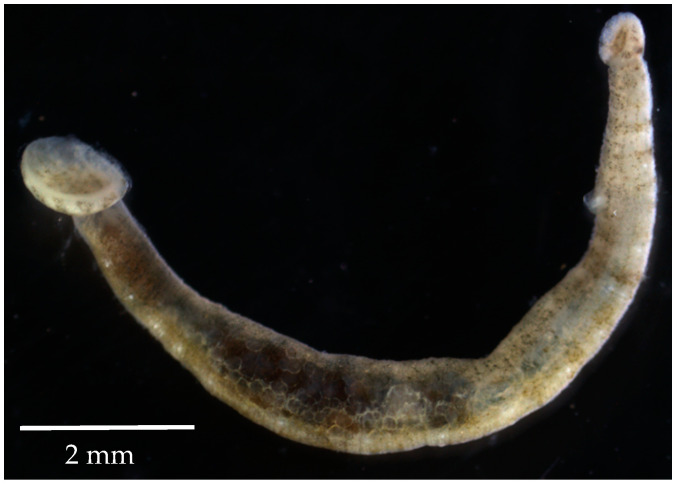
Parasitic leech *Zeylanicobdella arugamensis*.

Vibriosis is a bacterial disease that poses a significant threat to fish and shellfish in aquaculture systems. It is primarily caused by several species of *Vibrio* bacteria, including *V. parahaemolyticus*, *V. alginolyticus*, *V. anguillarum* and *V. harveyi*. Of these, *V. parahaemolyticus* stands out as one of the most dominant pathogens. These *Vibrio* bacteria are capable of infecting a wide range of fish species, such as sea bass, grouper, etc. [1,2]. Outbreaks of vibriosis in aquaculture farms are triggered by a combination of factors, including changes in the physicochemical properties of the water and overpopulation [3]. In addition to vibriosis, the aquaculture industry also has to deal with the problem of parasite infestation. A well-known example is the parasitic leech *Zeylanicobdella arugamensis* de Silva, 1963 (Hirudinea, Piscicolidae) (Figure 1), which thrives in tropical and subtropical regions along the coasts of several countries, including Malaysia, Thailand, Indonesia, Brunei Darussalam, Singapore, India, Iran, Sri Lanka, the Philippines, Australia and Japan [4,5,6,7]. Leong & Wong (1988) were the first to describe the infestations of unidentified parasitic leeches in Malaysian groupers cultured in floating cages, with a prevalence of 0.4 percent [8]. However, *Z. arugamensis* has also been regularly isolated in other marine-farmed fish [4,6,9,10], in addition to other parasites [11,12,13]. The parasitic leech has affected various farmed fish, including groupers, hybrid groupers, orange-spotted groupers, sea bass, etc. [6,9,10]. It attaches itself mainly in large numbers to the pectoral, ventral, anal and caudal fins, as well as to the skin folds under the lower jaw, around the eyes and in the mouth areas. Infected fish often show frayed fins, hemorrhages and larger wounds at the feeding and attachment site of the parasite [9]. Infected fish experience rapid mortality and the parasitic leeches are also thought to contribute to Vibriosis [5,9,14]. The mortality caused by *Vibrio* infections and these parasitic leeches has caused considerable damage to the aquaculture industry and resulted in significant economic losses [2,15].

In the aquaculture industry, the use of toxic chemicals is a common practice to control Vibriosis and prevent parasite infestations [2,16,17]. However, it is important to recognize that the use of these pollutants has serious negative impacts on fish, humans and the ecosystem as a whole [17,18]. It is therefore important to prioritize the development of biological control agents that effectively control Vibriosis and manage parasitic leeches.

Giant sword fern (GSF), known as *Nephrolepis biserrata*, is a medicinal plant belonging to the Nephrolepidaceae family and is locally known as “Paku Pedang”. The plant holds considerable potential as a source of metabolites with antiparasitic properties. In our previous studies, we investigated the antiparasitic potential of the methanol extract and fractions of the plant [6,19,20,21]. Building on this preliminary work, our current study aims to explore the antimicrobial potential of the aqueous extract of GSF and to identify several novel antibacterial and antiparasitic metabolites using an ultra-high-performance liquid chromatography–high-resolution mass spectrometry system (UHPLC-HRMS).

## 2. Results

### 2.1. Antivibrio Activity

The aqueous extract of GSF at a concentration of 100 mg/mL (0.5 mL) exhibited antivibrio activity against *V. parahaemolyticus* only compared to other *Vibrio* spp., including *V. alginolyticus*, *V. anguillarum* and *V. harveyi* (Table 1).

### 2.2. Antiparasitic Activity

Table 2 shows the antiparasitic activity of the aqueous extract of GSF. Complete parasite mortality was noticed after exposure to the various concentrations of the aqueous extracts. Higher concentrations of the aqueous extract (100 mg/mL) resulted in parasitic leeches’ mortality in a shorter time compared to medium (50 mg/mL) and low concentrations (25 mg/mL). On the other hand, no mortality was recorded in the control group treated with seawater only.

### 2.3. Physio-Chemical Parameters

Table 3 lists physio-chemical parameters, such as temperature, pH, salinity and dissolved oxygen. All the metrics are constant, except the pH values of the plant extracts, which differ slightly from the control groups.

### 2.4. Metabolites Detected in the Aqueous Extract of GSF

**Figure 2:**
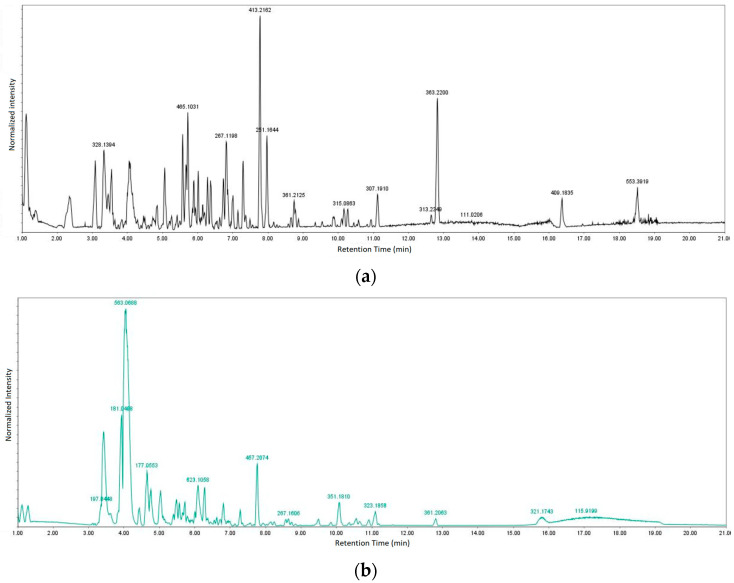
Chromatograms for (**a**) positive and (**b**) negative modes of data acquisition.

**Figure 3:**
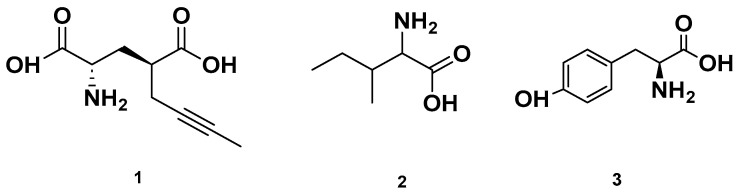
Chemical structures of amino acids detected in GSF.

**Figure 4:**
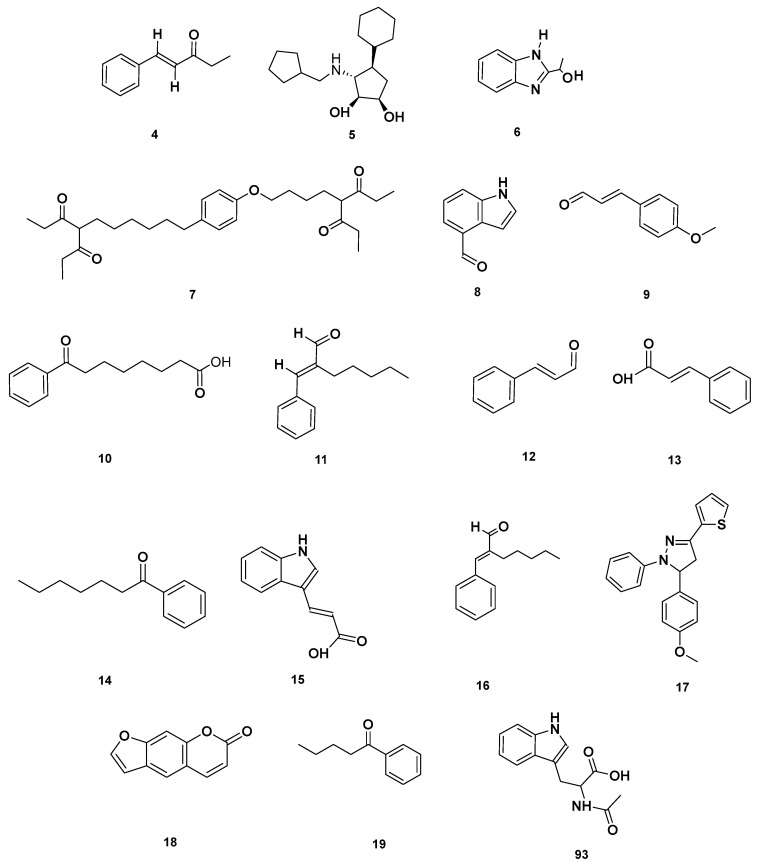
Aromatic compounds identified in GSF extracts.

**Figure 5:**
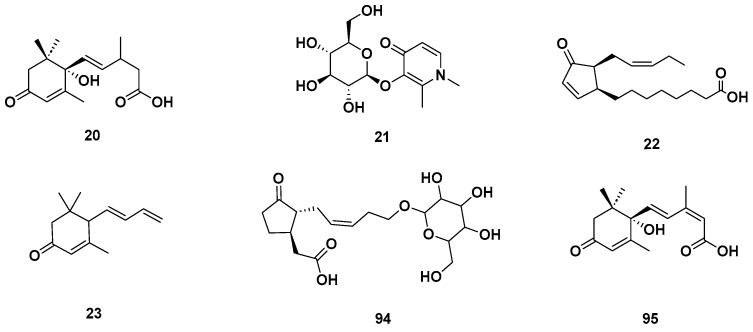
Cyclic ketones identified in GSF extracts.

**Figure 6:**
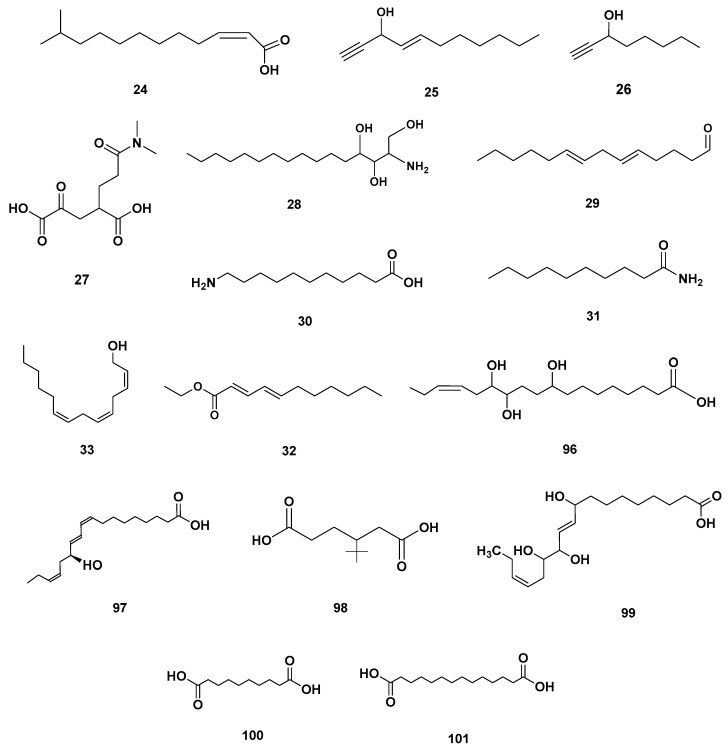
Fatty acyl identified in GSF extracts.

**Figure 7:**
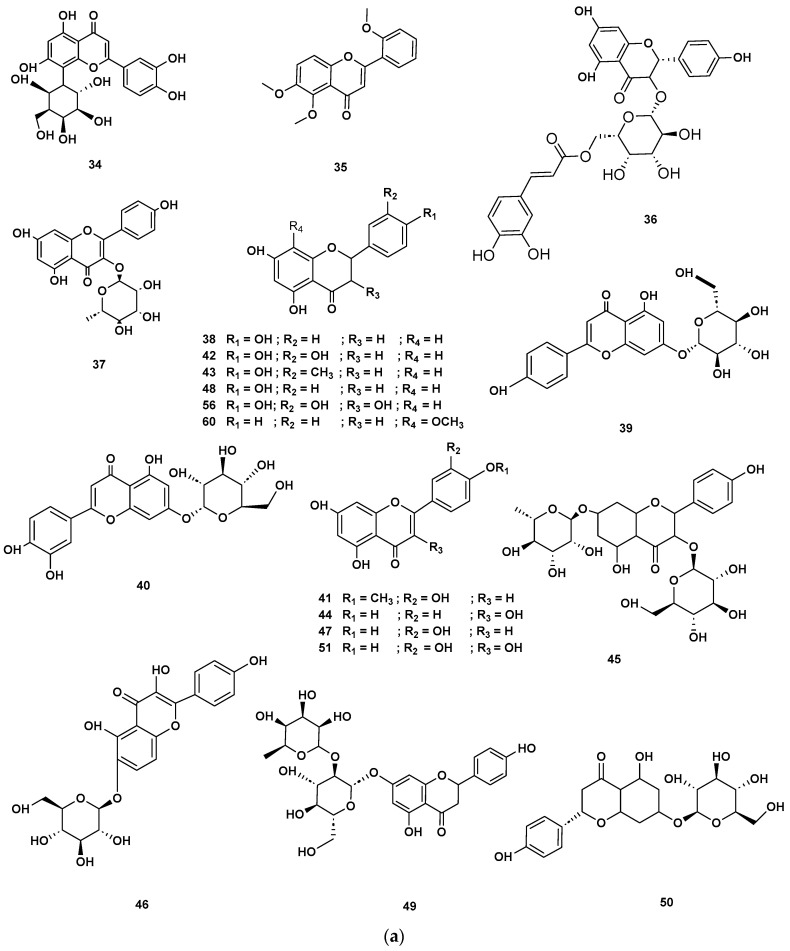

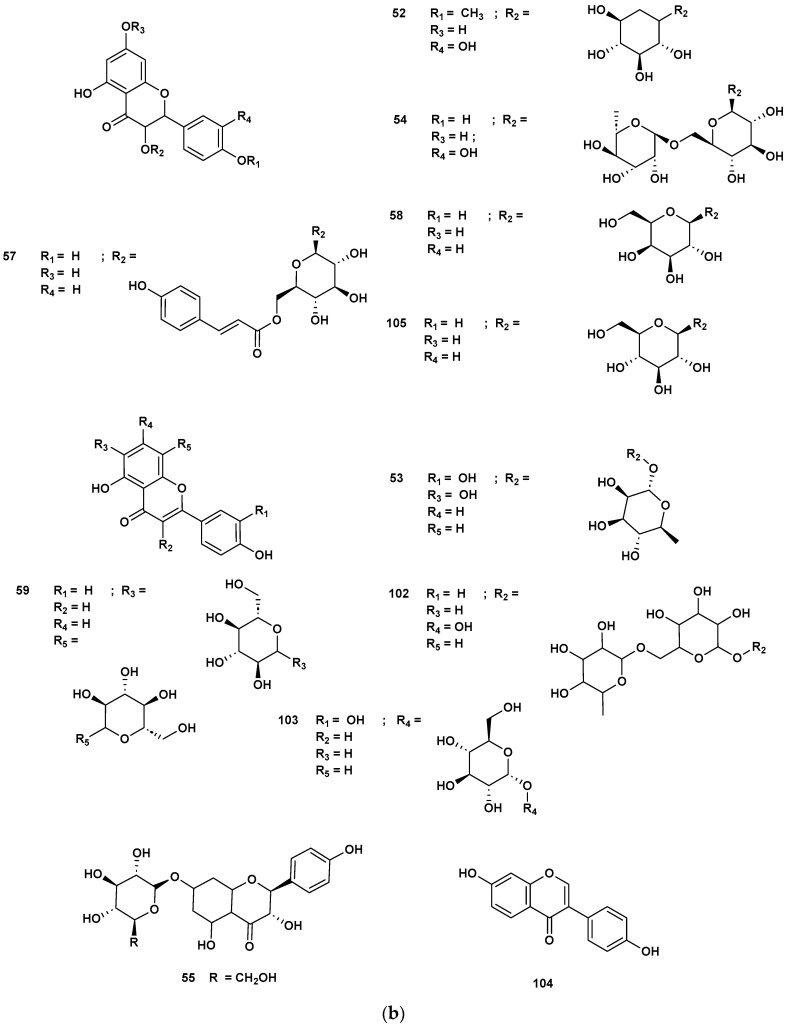
(**a**) Chemical structures of flavonoids identified in GSF extracts. (**b**) Chemical structures of additional flavonoids identified in GSF extracts.

**Figure 8:**
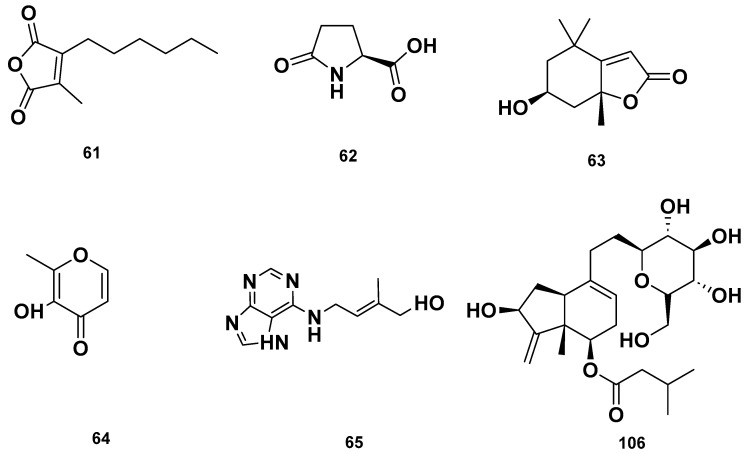
Heterocyclic compounds detected in GSF extracts.

**Figure 9:**
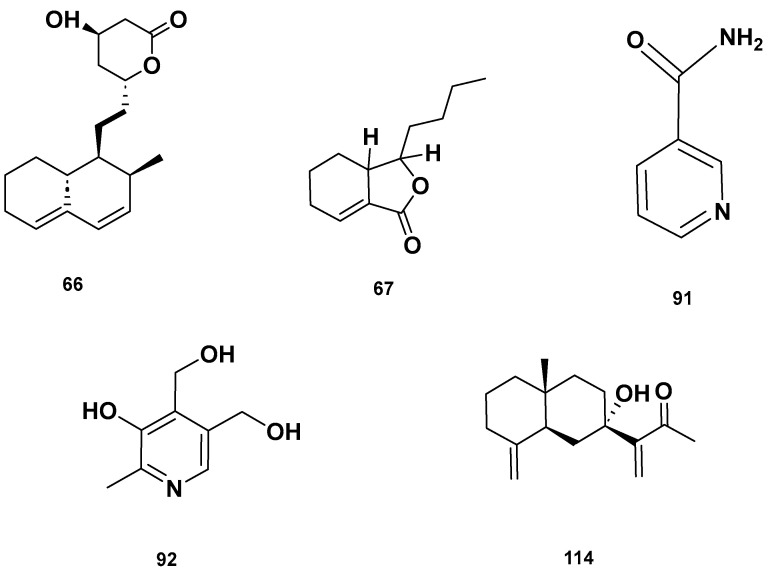
Lactone (**67**–**68**), Vitamin B (**92**–**93**) and polycyclic (**114**) metabolites in the GSF extracts.

**Figure 10:**
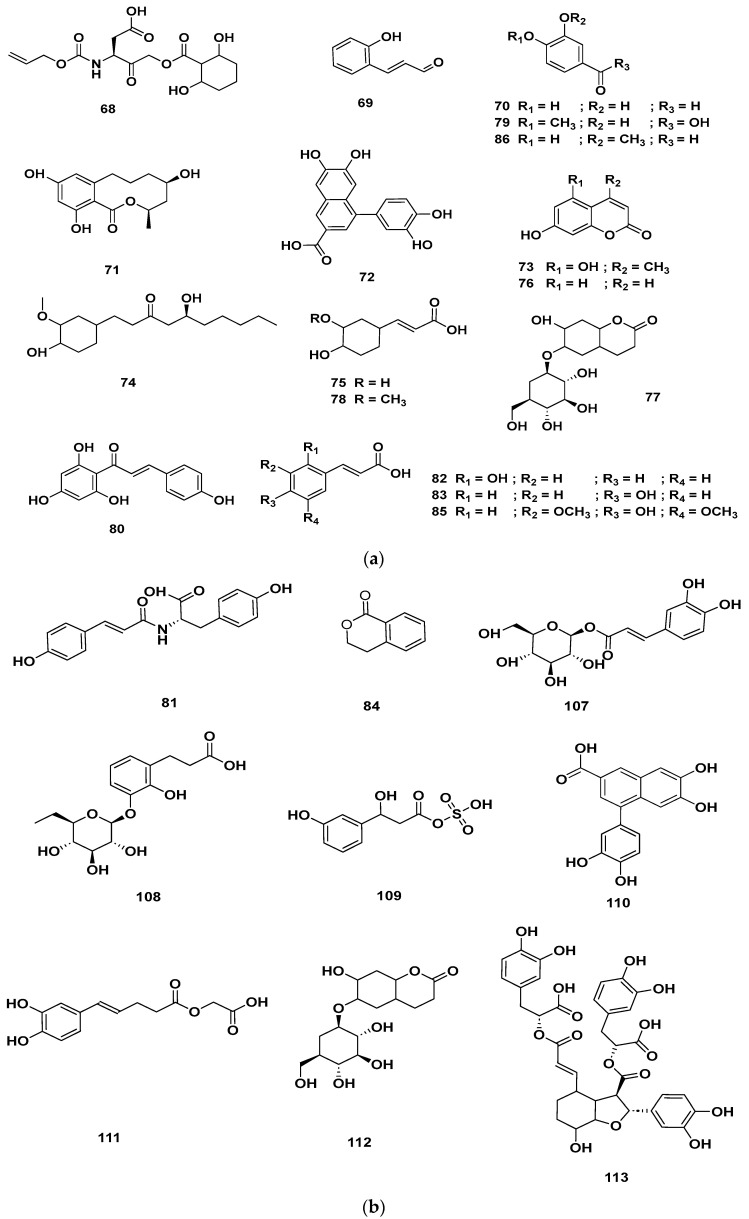
(**a**) Chemical structures of phenolics identified in GSF extracts. (**b**) Chemical structures of additional phenolics identified in GSF extracts.

**Figure 11:**
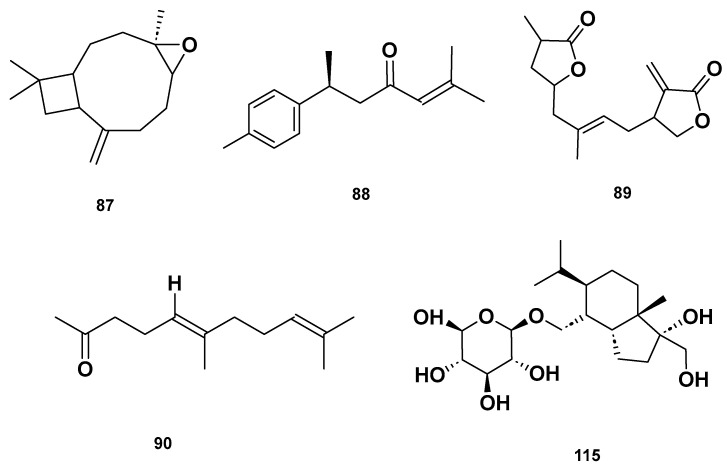
Chemical structures of terpenoids in the GSF extracts.

Table 4 and Table 5 list the names, molecular formulas and classes of metabolites identified in the aqueous extract of GSF using ultra-high-performance liquid chromatography–high-resolution mass spectrometry system (UHPLC-HRMS). The chromatograms for positive and negative modes of data acquisition are shown in Figure 2. Overall, 118 metabolites were effectively matched, with amino acids accounting for 3, aromatics for 18, cyclic ketones for 6, fatty acyl for 16, flavonoids for 32, heterocyclic for 6, lactone for 2, phenolics for 26, polycyclic for 1, steroid for 1, terpenoid for 5 and vitamin B2 for 2. The chemical structures of the metabolites are exhibited in Figure 3, Figure 4, Figure 5, Figure 6, Figure 7, Figure 8, Figure 9, Figure 10 and Figure 11 according to their respective chemical groups.

## 3. Discussion

Utilizing natural control agents to effectively combat bacterial infections and manage parasitic infestations is a viable and good alternative due to the existence of several beneficial metabolites [23]. In the current study, we aimed to test the aqueous extract of GSF against *Vibrio* spp., which is known to cause secondary infection in the infested host fish [5,9,14]. Our results displayed that the aqueous extract of GSF exhibited significant inhibition of *V. parahaemolyticus* at a 100 mg/mL concentration; however, no inhibition was noticed against *V. alginolyticus*, *V. anguillarum* and *V. harveyi*. This finding is consistent with previous research that has reported the antivibrio property of the methanol and chloroform extracts from the same plant against *V. parahaemolyticus* [24]. However, in the current study, the aqueous extract of GSF demonstrated enhanced antivibrio activity against *V. parahaemolyticus*, as evidenced by a larger diameter of the zone of inhibition measuring 19.5 mm. In comparison, the methanol and chloroform extracts have only displayed zone of inhibition measuring 8 and 6 mm, respectively [24]. Our results displayed that the aqueous extract of GSF possesses better antivibrio potential against *V. parahaemolyticus* compared to the previously examined methanol and chloroform extracts.

The aqueous extract of GSF also displayed antiparasitic potential and caused the complete death of the parasitic leech, *Z. arugamensis*, in a dose-dependent manner. At doses ranging from 100 to 25 mg/mL, all the parasitic leeches were killed in 11–43 min, whereas the methanol extract and fraction 3 at concentrations of 100 and 2.5 mg/mL of the same plant killed the leeches in an average time of 4 and 1.9 min, respectively [6,21]. It appears, based on our previously published data that the methanol extract of the plant caused parasitic leeches to die faster than the aqueous extract. On the other hand, the aqueous extract of GSF revealed different metabolites compared to the methanol extract and fractions. The methanol and aqueous extracts of the neem plant have been reported to have antiparasitic activity against *Z. arugamensis*. The extract, at a concentration of 100 mg/mL, has resulted in the mortality of parasitic leeches within a time limit of 9 and 6 min, respectively. The methanol leaves extract of *Dillenia suffruticosa* at concentrations of 100 mg/mL has resulted in the complete mortality of parasitic leeches in 14 [25]. The aqueous extracts of some of the tropical plants, such as *Melastoma malabathricum*, *Piper betle*, *Tetracera indica* and *Etlingera coccinea*, have been tested in vitro for potential anti-leech *Z. arugamensis* activity. The anti-leech activity has been determined by exposing *Z. arugamensis* to 20 μL of plant extract (500 mg/mL) for 5 min in a 24-well plate. The effects of aqueous extracts of all the mentioned plants on the leeches were very rapid, causing death in less than 5 min [26]. The published data indicated that plant extracts can be applied as a natural control against parasitic leeches. In addition, plant extracts have also been reported to have antiparasitic activity against other classes of parasitic leeches, which include *Allium sativum* against the leech *Limnatis nilotica*. The methanol extract of *A. sativum* at concentrations of 600 µg/mL resulted in the death of leeches in 68 min [27]. The anti-leech effects of methanol extracts of *Cassia alata*, *Costus afer*, *Ficus sur* and *Platostoma africanum* have been reported against the parasitic leech *Hirudo medicinalis* [28].

The UHPLC-HRMS analysis displayed the metabolic composition of the aqueous extract of GSF. This analysis enabled us to identify several classes of metabolites, including flavonoids, phenolics, fatty acyls, etc. Notably, flavonoids and phenolics were found to be the dominant metabolites in the extracts. These classes of metabolites have been identified in the extract of other plant species and have been associated with antioxidant, antimicrobial, anti-inflammatory and antiparasitic potential [29,30,31,32,33,34,35]. However, we have not detected any peptides or large lipids in the aqueous extract of our sample, which might be attributed to the aqueous extract’s lower affinity for peptides and lipids in comparison to methanol extraction. In contrast, our analysis of the aqueous extract of GSF revealed more metabolites compared to our previous examination of the methanol extract [6]. This demonstrates that the extraction efficiency of GSF with the aqueous solution is higher than that of methanol, an enhancement likely due to the boiling effect. Additionally, our profiling was specifically targeted within the 100–1500 *m/z* range.

Metabolites, such as abscisic acid [36], daidzein [37], quercetin-3β-D-glucoside [38] and salvianolic acid B [39,40], have been reported to possess antibacterial properties. Among the amino acids identified in the aqueous extracts, isoleucine and tyrosin were prominent. These amino acids play an important role as antioxidant agents [30,31]. Additionally, aromatic compounds, such as cinnamaldehyde and cinnamic acid, have been found to possess antiparasitic activity against *Dactylogyrus intermedius* monogenean parasites [29]. Flavonoid compounds, including apigenin and quercetin, have been found to possess antiparasitic efficacy against *Leishmania tropica* amastigotes, *Cryptosporidium parvum* and *Encephalitozoon intestinalis* [32,33]. Cynaroside has been found to possess antiparasitic properties against the protozoan *Leishmania donovani* [41], while eriodictyol and kaempferol have shown antiparasitic activity against *Plasmodium falciparum* and *P. berghei* [42,43]. Luteolin has been reported to display antiparasitic activity against *L. donovani* [44], and naringenin has shown promise in combating *Eimeria* spp. Parasite [45]. Wogonin has also exhibited antiparasitic activity against *Schistosoma mansoni* [46]. Furthermore, phenolic compounds, like 6-gingerol in combination with amphotericin B, have been found to have antiparasitic potential against *Leishmania infection* [47]. Nicotinamide, a type of vitamin B, has demonstrated antiparasitic activities against *P. falciparum*, *Trypanosoma cruzi.*

The presence of these antibacterial and antiparasitic metabolites in the aqueous extract of GSF suggests that they may be responsible for the inhibition of *Vibrio* spp. and the elimination of parasitic leeches. However, it is essential to conduct further research to isolate these antibacterial and antiparasitic metabolites and access their efficacy in their pure form against the *Vibrio* spp. and parasitic leeches.

Furthermore, our research also revealed that the aqueous extract of GSF contained more diverse metabolites compared to the previously reported methanol extract of the same plants, particularly in terms of flavonoid and phenolic metabolites [6].

## 4. Materials and Methods

### 4.1. Sample Collection and Extraction

The aerial parts of the plant were taken from Universiti Malaysia Sabah. The plant was recognized and a voucher specimen was deposited at Universiti Malaysia Sabah, Kota Kinabalu. The leaves of the plant were cleaned with distilled water and oven dried at 37 °C. A high-capacity mill was used to grind the dried plant separately. In total, 100 g of the dry plant powder was boiled with distilled water in a ratio of 1:10 for 10 min on a stir plate. The decoctions were drawn off and allowed to cool at room temperature for 1 h. The extracts were filtered through a strainer to remove coarse residues, and the filtrate was then filtered again using Whatman No. 1 filter paper. The filtrate was lyophilized using a freeze dryer after being held at −80 °C for 24 h.

### 4.2. Vibrio Strains and Stock Preparation

The antivibrio activities of the extract were tested against four different species of Gram-negative bacteria of the genus *Vibrio*, including *V. alginolyticus* (ATCC17749), *V. anguillarum* (ATCC19264), *V. harveyi* (ATCC35084) and *V. parahaemolyticus* (ATCC17802). The bacterial stock was obtained from the Fish Disease Laboratory, Borneo Marine Research Institute, Universiti Malaysia Sabah. The bacterial strains were thawed and cultured. Approximately 50 µL of the bacteria were cultured in 5 mL tryptone soy broth (TSB) containing 2% sodium chloride and incubated for 24 h at 25 °C in a shaking incubator.

### 4.3. Antivibrio Activity

The antivibrio activity of the extract was evaluated using the disc diffusion method with slight modifications [48]. Tryptone soy agar plates were used in a biosafety chamber to simulate aseptic conditions and prevent contamination. The agar plates were divided into two sections to test the same extract at the same concentration. Whatman filter discs (8 mm diameter) were inserted into the nutrient agar plates using a sterile cork borer (5 mm), and the bacteria were spread on the solid plates using a sterile swab moistened with the bacterial suspension. Then 100 μL of the aqueous extract (100 mg/mL) was added to the discs prepared in the inoculated plates. As a positive control, filter paper discs containing oxytetracycline (10 µg in 2 mL DMSO) were utilized. The plates were incubated at 37 °C for 24 h, and the zone of inhibition, if any, around the wells was measured in mm.

### 4.4. Parasitic Leech Collection

The parasitic leeches, *Z. arugamensis*, ranging in length from 1 to 1.5 cm, were obtained from the aquaculture facilities of Borneo Marine Research Institute, Universiti Malaysia Sabah. A hybrid grouper (*Epinephelus fuscoguttatus × E. lanceolatus*) (20–350 g) infested with parasitic leeches were placed in a small tank containing seawater from the cage, and leeches were removed individually by hand. The separated leeches were placed into a container containing filtered seawater.

### 4.5. Antiparasitic Activity

Healthy and adult parasitic leeches were selected and divided into 5 groups. Each group was provided with 6 parasitic leeches in a Petri dish.

Group 1 was treated with seawater only (negative control).

Group 2 was treated with 0.25% formalin solution (positive control).

Group 3 was treated with 25, mg/mL of the aqueous extract of GSF.

Group 4 was treated with 50, mg/mL of the aqueous extract of GSF.

Group 5 was treated with 100, mg/mL of the aqueous extract of GSF, respectively.

The aqueous extract was dissolved in filtered seawater and applied to the parasitic leeches in a Petri dish. During the experiment, inactivity, mortality time and percentage of the parasite leeches were recorded [21].

### 4.6. Liquid Chromatography

Liquid chromatography (LC) was performed using a Dionex UltiMate 3000 UHPLC system (Thermo Fisher Scientific, Waltham, MA, USA) coupled to a Thermo Syncronis C18 column (2.1 mm × 100 mm × 1.7 μm; Thermo Fisher Scientific, Waltham, MA, USA), which was maintained at 55 °C and a flow rate of 450 µL/min during analysis. The mobile phases consisted of solvent A (water added with 0.1% formic acid) and solvent B (acetonitrile added with 0.1% formic acid). The gradient elution program was started at 0.5% of solvent B for 1 min, then from 0.5% to 99.5% of solvent B for 15 min and held for 4 min. The columns were then conditioned for 2 min before the next injection. The injection volume was set to 2 µL.

### 4.7. Data Acquisition

MS and MS/MS data were acquired using the Thermo Scientific Q Exactive HF Orbitrap mass spectrometry system (Thermo Fisher Scientific, Waltham, MA, USA), which is equipped with a heated electrospray ionization (HESI) probe. Data acquisition was set between an *m*/*z* of 100–1000 for MS and 200–1000 for MS /MS. The resolutions of the MS and MS/MS data were acquired at 60k and 15k, respectively. Positive and negative HESI were both used at 3.5 kV and 3.0 kV, respectively. Ion source conditions were set as follows: capillary temperature of 320 °C, sheath gas flow rate of 50, the aux gas flow rate of 18, sweep gas flow rate of 0 and aux gas heater temperature of 300 °C. Calibrations were performed with Pierce LTQ ESI Positive Calibrations solution and Pierce LTQ ESI Negative Calibrations solution (Thermo Fisher Scientific, Waltham, MA, USA) before analyzing the samples.

### 4.8. Data Analysis

The recorded data were processed and analyzed using Thermo Scientific Compound Discoverer 3.0 software (Thermo Fisher Scientific, Waltham, MA, USA) with the default settings for compound identification. Briefly, the default workflow includes background subtraction with blank data, retention time alignment, feature detection, elemental composition determination, library matching and fragment ion search (FISh) scoring. Compound identification was primarily based on matching MS/MS data with the mzCloud and mzVault databases. Mismatched signals were attempted with the ChemSpider database using MS data and supported with a FISh scoring of above 50.

### 4.9. Statistical Analysis

The IBM SPSS Statistics 25 Window package (IBM, Armonk, NY, US) was used to analyze the data. Significant differences between groups were determined using one-way analysis of variance (ANOVA) followed by Tukey’s multiple comparison tests. The means and standard error of the means (S.E.) were used to present all results. The *p*-values of less than 0.05 were considered significant.

## 5. Conclusions

The results of the current study are very promising, highlighting the potential of the aqueous extract of giant sword fern (GSF) in inhibiting *Vibrio* spp., especially *V. parahemlyticus*, and eliminating parasitic leeches. Analysis using the ultra-high-performance liquid chromatography–high-resolution mass spectrometry system revealed the presence of 118 compounds, with flavonoid metabolites accounting for the majority, followed by phenolic, aromatic, fatty acyl, terpenoid, vitamin and steroidal metabolites. Cinnamaldehyde, cinnamic acid, apigenin, quercetin, cynaroside, luteolin, naringenin, wogonin, 6-gingerol, nicotinamide, abscisic acid, daidzein, querce-tin-3β-d-glucoside, salvianolic acid B and others are examples of metabolites with antibacterial and antiparasitic activity. The results suggest that the aqueous extract of GSF can be used as a natural alternative to control *Vibrio* infections and to treat parasitic leeches in aquaculture systems.

## Figures and Tables

**Table 1:** Antivibrio activity of aqueous extract of GSF.

No.	Groups	Diameter of Inhibition Zone (mm) Mean ± S.D
1	Oxytetracycline (positive control)	55.0 ± 1.0
2	*V. alginolyticus*	0
3	*V. anguillarum*	0
4	*V. harveyi*	0
5	*V. parahaemolyticus*	19.5 ± 2.50 *

Each value represents the mean ± S.D (*n* = 3). * Significance at *p* < 0.05 compared with the control group.

**Table 2:** Time till death and percentage of all parasitic leeches at different concentrations of GSF aqueous extract.

No.	Group	Time Till Death (mins) Mean ± S.D	Death Percentage
1	Normal control	>720.00 ± 00	0
2	Positive control (Formalin 0.25%) (*v*/*v*)	3.62 ± 0.42 *	100
3	GSF aqueous extract (25 mg/mL)	43.89 ± 3.97 *#	100
4	GSF aqueous extract (50 mg/mL)	27.26 ± 4.65 *#$	100
5	GSF aqueous extract (100 mg/mL)	11.30 ± 2.42 *#$^	100

Each value represents the mean ± S.D of 6 parasitic leeches per group. * Significance at *p* < 0.05 compared with the control group. # Significance at *p* < 0.05 compared with the formalin-treated group (0.25% *v*/*v*). $ Significance at *p* < 0.05 compared with GSF aqueous extract (25 mg/mL). ^ Significance at *p* < 0.05 compared with GSF aqueous extract (50 mg/mL).

**Table 3:** Water quality parameters of the solutions of the normal control and treatment groups.

No.	Water Parameters		Concentrations			
	Groups	Normal Control	Positive Control (Formalin 0.25%) (*v*/*v*)	GSF Aqueous Extract (mg/mL)		
				(25)	(50)	(100)
1	Temperature (°C)	25.6	25.9	25.9	24.9	24.9
2	pH	7.77	7.30	5.59	5.38	5.08
3	Salinity (ppt)	30.0	30.9	31	31	31
4	Dissolved oxygen (mg/L)	7.2	6.7	7.0	7.1	7.1

**Table 4:** Metabolite profiles in aqueous extract of GSF analyzed via positive mode UHPLC-HRMS.

Name	R. Time (mins)	Formula	Mass Error (ppm)	Calc. Molecular Mass	Database	Matching Score *	Class
(4*R*)-4-but-2-yn-1-yl-l-glutamic acid (**1**)	3.45	C_9_H_13_NO_4_	0.01	199.0845	ChemSpider	63.3	Amino acid
Isoleucine (**2**)	1.43	C_6_H_13_NO_2_	1.11	131.0948	mzCloud	99.1	Amino acid
Tyrosine (**3**)	1.07	C_9_H_11_NO_3_	1.05	181.0741	mzCloud	99.0	Amino acid
(1*E*)-1-Phenyl-1-penten-3-one (**4**)	7.73	C_11_H_12_O	−0.14	160.0888	ChemSpider	60.0	Aromatic
(1*R*,2*S*,3*R*,4*R*)-3-[(Cyclopentylmethyl)amino]-4-phenyl-1,2-cyclopentanediol (**5**)	10.01	C_17_H_25_NO_2_	−0.26	275.1885	mzCloud	87.0	Aromatic
1-(1*H*-Benzo[d]imidazol-2-yl)ethan-1-ol (**6**)	3.02	C_9_H_10_N_2_O	0.50	162.0794	mzCloud	81.0	Aromatic
4-(4-{4-[(7-oxo-6-Propionylnonyl)oxy] phenoxy}butyl)-3,5-heptanedione (**7**)	7.52	C_29_H_44_O_6_	1.08	488.3143	ChemSpider	56.8	Aromatic
4-Indolecarbaldehyde (**8**)	6.12	C_9_H_7_NO	−0.40	145.0527	mzCloud	94.7	Aromatic
4-Methoxycinnamaldehyde (**9**)	5.38	C_10_H_10_O_2_	−0.71	162.0680	ChemSpider	52.4	Aromatic
7-Benzoylheptanoic acid (**10**)	7.61	C_14_H_18_O_3_	−0.14	234.1256	ChemSpider	73.7	Aromatic
Amylcinnamaldehyde (**11**)	7.46	C_14_H_18_O	0.88	202.1359	ChemSpider	66.0	Aromatic
Cinnamaldehyde (**12**)	4.37	C_9_H_8_O	0.21	132.0575	ChemSpider	60.0	Aromatic
Cinnamic acid (**13**)	7.13	C_9_H_8_O_2_	0.41	148.0525	ChemSpider	50.0	Aromatic
Heptanophenone (**14**)	6.60	C_13_H_18_O	0.45	190.1359	ChemSpider	69.8	Aromatic
Indole-3-acrylic acid (**15**)	5.67	C_11_H_9_NO_2_	−0.01	187.0633	mzCloud	98.3	Aromatic
Jasmonal (**16**)	11.62	C_14_H_18_O	0.65	202.1359	ChemSpider	73.7	Aromatic
*N*-{2-[(4-methylphenyl)thio]pyridin-3-yl}-2-phenylacetamide (**17**)	4.82	C_20_H_18_N_2_OS	−0.13	334.1139	mzCloud	90.9	Aromatic
Psoralen (**18**)	4.77	C_11_H_6_O_3_	0.13	186.0317	mzCloud	81.5	Aromatic
Valerophenone (**19**)	6.81	C_11_H_14_O	0.13	162.1045	ChemSpider	53.6	Aromatic
Abscisic acid (**20**)	10.09	C_15_H_20_O_4_	−0.70	264.1360	mzCloud	82.7	Cyclic ketone
1,2-Dimethyl-4-oxo-1,4-dihydro-3-pyridinyl β-d-glucopyranoside (**21**)	4.28	C_13_H_19_NO_7_	−0.25	301.1161	ChemSpider	52.9	Cyclic ketone
12-oxo Phytodienoic acid (**22**)	8.15	C_18_H_28_O_3_	−0.47	292.2037	ChemSpider	75.8	Cyclic ketone
4-(1,3-Butadienyl)-3,5,5-trimethylcyclohex-2-en-1-one (**23**)	5.00	C_13_H_18_O	0.38	190.1358	ChemSpider	51.4	Cyclic ketone
(2*Z*)-11-Methyl-2-dodecenoic acid (**24**)	6.06	C_13_H_24_O_2_	−0.15	212.1776	ChemSpider	80.8	Fatty acyl
(4*E*)-4-Undecen-1-yn-3-ol (**25**)	4.87	C_11_H_18_O	0.15	166.1358	ChemSpider	69.4	Fatty acyl
1-Octyn-3-ol (**26**)	4.84	C_8_H_14_O	1.88	126.1047	ChemSpider	52.6	Fatty acyl
2-[3-(Dimethylamino)-3-oxopropyl]-4-oxopentanedioic acid (**27**)	2.99	C_10_H_15_NO_6_	−0.31	245.0899	ChemSpider	82.1	Fatty acyl
2-Amino-1,3,4-octadecanetriol (**28**)	21.50	C_18_H_39_NO_3_	0.03	317.2930	mzCloud	82.8	Fatty acyl
5,8-Tetradecadienal (**29**)	10.66	C_14_H_24_O	−0.17	208.1827	ChemSpider	65.4	Fatty acyl
9-Aminononanoic acid (**30**)	4.31	C_9_H_19_NO_2_	0.95	173.1417	ChemSpider	53.5	Fatty acyl
Decanamide (**31**)	10.02	C_10_H_21_NO	0.69	171.1624	mzCloud	84.8	Fatty acyl
Ethyl (2*E*,4*E*)-2,4-undecadienoate (**32**)	5.38	C_13_H_22_O_2_	0.92	210.1622	ChemSpider	54.4	Fatty acyl
Tetradeca-2*Z*,5*Z*,8*Z*-trien-1-ol (**33**)	10.58	C_14_H_24_O	−0.17	208.1827	ChemSpider	71.2	Fatty acyl
2-(3,4-Dihydroxyphenyl)-8-galactopyranosyl-5,7-dihydroxy-4*H*-1-benzopyran-4-one (**34**)	5.26	C_21_H_20_O_11_	−0.04	448.1005	mzCloud	85.8	Flavonoid
5,6,2’-Trimethoxyflavone (**35**)	10.16	C_18_H_16_O_5_	−0.74	312.0995	mzCloud	88.4	Flavonoid
5,7-Dihydroxy-2-(4-hydroxyphenyl)-4-oxo-4*H*-chromen-3-yl 6-*O*-[(2*E*)-3-(3,4-dihydroxyphenyl)-2-propenoyl]-β-d-galactopyranoside (**36**)	6.82	C_30_H_26_O_14_	0.39	610.1325	ChemSpider	68.6	Flavonoid
Afzelin (**37**)	6.59	C_21_H_20_O_10_	0.19	432.1057	mzCloud	95.2	Flavonoid
Apigenin (**38**)	5.84	C_15_H_10_O_5_	−0.38	270.0527	mzCloud	99.5	Flavonoid
Cosmosiin (**39**)	5.84	C_21_H_20_O_10_	−0.09	432.1056	mzCloud	99.1	Flavonoid
Cynaroside (**40**)	5.42	C_21_H_20_O_11_	−0.45	448.1004	mzCloud	81.2	Flavonoid
Diosmetin (**41**)	6.06	C_16_H_12_O_6_	−0.87	300.0631	mzCloud	96.0	Flavonoid
Eriodictyol (**42**)	5.64	C_15_H_12_O_6_	−0.59	288.0632	mzCloud	98.4	Flavonoid
Homoeriodictyol (**43**)	6.34	C_16_H_14_O_6_	−0.44	302.0789	mzCloud	86.3	Flavonoid
Kaempferol (**44**)	5.98	C_15_H_10_O_6_	0.14	286.0478	mzCloud	99.1	Flavonoid
Kaempferol-3-*O*-β-glucopyranosyl-7-*O*-α-rhamnopyranoside (**45**)	5.54	C_27_H_30_O_15_	0.61	594.1588	mzCloud	98.9	Flavonoid
Kaempferol-7-*O*-glucoside (**46**)	4.95	C_21_H_20_O_11_	0.23	448.1007	mzCloud	99.3	Flavonoid
Luteolin (**47**)	5.42	C_15_H_10_O_6_	−0.18	286.0477	mzCloud	97.6	Flavonoid
Naringenin (**48**)	6.15	C_15_H_12_O_5_	−0.31	272.0684	mzCloud	94.4	Flavonoid
Naringin (**49**)	6.08	C_27_H_32_O_14_	0.81	580.1797	mzCloud	94.4	Flavonoid
Prunin (**50**)	6.15	C_21_H_22_O_10_	0.49	434.1215	mzCloud	99.0	Flavonoid
Quercetin (**51**)	5.72	C_15_H_10_O_7_	−0.61	302.0425	mzCloud	99.4	Flavonoid
Quercetin-3β-d-glucoside (**52**)	5.72	C_21_H_20_O_12_	−0.08	464.0954	mzCloud	98.2	Flavonoid
Quercitrin (**53**)	6.16	C_21_H_20_O_11_	0.43	448.1008	mzCloud	89.3	Flavonoid
Rutin (**54**)	5.58	C_27_H_30_O_16_	0.55	610.1537	mzCloud	99.1	Flavonoid
Sinensin (**55**)	4.79	C_21_H_22_O_11_	0.38	450.1164	mzCloud	85.6	Flavonoid
Taxifolin (**56**)	4.36	C_15_H_12_O_7_	−0.09	304.0583	mzCloud	93.5	Flavonoid
Tiliroside (**57**)	7.23	C_30_H_26_O_13_	0.47	594.1376	mzCloud	98.9	Flavonoid
Trifolin (**58**)	5.98	C_21_H_20_O_11_	0.54	448.1008	mzCloud	84.3	Flavonoid
Vicenin-2 (**59**)	4.87	C_27_H_30_O_15_	1.48	594.1594	mzCloud	88.0	Flavonoid
Wogonin (**60**)	10.12	C_16_H_12_O_5_	−0.02	284.0685	mzCloud	88.2	Flavonoid
3-Hexyl-4-methyl-2,5-furandione (**61**)	6.18	C_11_H_16_O_3_	0.76	196.1101	ChemSpider	51.9	Heterocyclic
D-(+)-Pyroglutamic Acid (**62**)	1.12	C_5_H_7_NO_3_	1.15	129.0427	mzCloud	96.6	Heterocyclic
Loliolide (**63**)	6.07	C_11_H_16_O_3_	0.76	196.1101	ChemSpider	61.1	Heterocyclic
Maltol (**64**)	3.84	C_6_H_6_O_3_	0.85	126.0318	mzCloud	99.4	Heterocyclic
Zeatin (**65**)	3.36	C_10_H_13_N_5_O	0.17	219.1121	mzCloud	89.3	Heterocyclic
(4*R*,6*R*)-4-Hydroxy-6-{2-[(1*S*,2*S*,8a*R*)-2-methyl-1,2,6,7,8,8a-hexahydro-1-naphthalenyl]ethyl}tetrahydro-2*H*-pyran-2-one (**66**)	11.62	C_18_H_26_O_3_	−0.23	290.1881	ChemSpider	62.5	Lactone
Sedanolide (**67**)	5.76	C_12_H_18_O_2_	0.70	194.1308	ChemSpider	56.4	Lactone
(3*S*)-3-{[(Allyloxy)carbonyl]amino}-5-[(2,6-dihydroxybenzoyl)oxy]-4-oxopentanoic acid (**68**)	3.67	C_16_H_17_NO_9_	−0.28	367.0902	ChemSpider	55.6	Phenolic
2-Hydroxycinnamaldehyde (**69**)	3.08	C_9_H_8_O_2_	0.13	148.0525	ChemSpider	64.3	Phenolic
3,4-Dihydroxybenzaldehyde (**70**)	4.10	C_7_H_6_O_3_	0.11	138.0317	mzCloud	85.5	Phenolic
3*R*,5*R*-Sonnerlactone (**71**)	11.00	C_14_H_18_O_5_	−0.65	266.1153	ChemSpider	57.9	Phenolic
4-(3,4-Dihydroxyphenyl)-6,7-dihydroxy-2-naphthoic acid (**72**)	3.47	C_17_H_12_O_6_	−1.15	312.0630	mzCloud	97.0	Phenolic
5,7-Dihydroxy-4-methylcoumarin (**73**)	5.02	C_10_H_8_O_4_	0.69	192.0424	mzCloud	93.8	Phenolic
6-Gingerol (**74**)	9.71	C_17_H_26_O_4_	−0.88	294.1829	ChemSpider	59.8	Phenolic
Caffeic acid (**75**)	4.06	C_9_H_8_O_4_	−0.34	180.0422	mzCloud	98.8	Phenolic
Esculetin (**76**)	4.15	C_9_H_6_O_4_	−0.32	178.0266	mzCloud	99.0	Phenolic
Esculin (**77**)	4.50	C_15_H_16_O_9_	−0.65	340.0792	mzCloud	94.4	Phenolic
Ferulic acid (**78**)	5.04	C_10_H_10_O_4_	0.32	194.0580	mzCloud	95.9	Phenolic
Isovanillic acid (**79**)	3.64	C_8_H_8_O_4_	0.70	168.0424	mzCloud	83.9	Phenolic
Naringeninchalcone (**80**)	6.07	C_15_H_12_O_5_	−0.02	272.0685	mzCloud	96.3	Phenolic
*N*-p-Coumaroyltyrosine (**81**)	6.09	C_18_H_17_NO_5_	−0.56	327.1105	mzCloud	99.3	Phenolic
o-Coumaric acid (**82**)	5.12	C_9_H_8_O_3_	0.49	164.0474	mzCloud	96.8	Phenolic
p-Coumaric acid (**83**)	4.67	C_9_H_8_O_3_	0.53	164.0474	mzCloud	80.6	Phenolic
Phyllodulcin (**84**)	11.12	C_9_H_8_O_2_	−0.30	148.0524	ChemSpider	55.0	Phenolic
Sinapinic acid (**85**)	4.56	C_11_H_12_O_5_	1.03	224.0687	mzCloud	94.7	Phenolic
Vanillin (**86**)	5.42	C_8_H_8_O_3_	0.64	152.0474	mzCloud	90.5	Phenolic
(-)-Caryophyllene oxide (**87**)	7.54	C_15_H_24_O	−0.09	220.1827	mzCloud	86.5	Terpenoid
(+)-ar-Turmerone (**88**)	6.74	C_15_H_20_O	0.92	216.1516	ChemSpider	76.7	Terpenoid
*epi*-Antheindurolide A (**89**)	4.56	C_15_H_20_O_4_	0.57	264.1363	mzCloud	89.6	Terpenoid
Geranylacetone (**90**)	6.06	C_13_H_22_O	0.10	194.1671	ChemSpider	78.1	Terpenoid
Nicotinamide (**91**)	1.10	C_6_H_6_N_2_O	1.69	122.0482	mzCloud	94.0	Vitamin B
Pyridoxine (**92**)	1.08	C_8_H_11_NO_3_	1.41	169.0741	mzCloud	96.4	Vitamin B
Steroidal compound	6.02	C_27_H_44_O_7_	−1.01	480.3082	mzCloud	94.1	Steroid

* Matching score: MS/MS Score (MzCloud)/FISh Score (ChemSpider).

**Table 5:** Secondary metabolite profiles in aqueous extract of GSF analyzed via negative mode UHPLC-HRMS.

Name	R. Time (mins)	Formula	Mass Error (ppm)	Calc. Molecular Mass	Database	Matching Score *	Class
*N*-Acetyltryptophan (**93**)	5.69	C_13_H_14_N_2_O_3_	0.20	246.1005	mzCloud	98.9	Aromatic
{(1*R*,2*R*)-2-[(2*Z*)-5-(Hexopyranosyloxy)-2-penten-1-yl]-3-oxocyclopentyl}acetic acid (**94**)	4.70	C_18_H_28_O_9_	0.29	388.1734	mzCloud	87.8	Cyclic ketone
Abscisic acid (**95**)	9.52	C_15_H_20_O_4_	0.67	264.1363	mzCloud	81.2	Cyclic ketone
(15*Z*)-9,12,13-Trihydroxy-15-octadecenoic acid (**96**)	8.60	C_18_H_34_O_5_	0.34	330.2407	mzCloud	92.8	Fatty acyl
(9*Z*,11*E*,13*S*,15*Z*)-13-Hydroxy-9,11,15-octadecatrienoic acid (**97**)	11.69	C_18_H_30_O_3_	0.42	294.2196	mzCloud	82.7	Fatty acyl
3-tert-Butyladipic acid (**98**)	7.20	C_10_H_18_O_4_	−1.72	202.1202	mzCloud	83.7	Fatty acyl
Corchorifatty acid F (**99**)	8.17	C_18_H_32_O_5_	0.98	328.2253	mzCloud	97.8	Fatty acyl
Dodecanedioic acid (**100**)	6.77	C_12_H_22_O_4_	−0.93	230.1516	mzCloud	97.1	Fatty acyl
Tetradecanedioic acid (**101**)	10.12	C_14_H_26_O_4_	−0.08	258.1831	mzCloud	85.0	Fatty acyl
5,7-Dihydroxy-2-(4-hydroxyphenyl)-4-oxo-4*H*-chromen-3-yl 6-*O*-(6-deoxyhexopyranosyl)hexopyranoside (**102**)	5.97	C_27_H_30_O_15_	1.42	594.1593	mzCloud	83.3	Flavonoid
Cynaroside (**103**)	6.01	C_21_H_20_O_11_	0.49	448.1008	mzCloud	82.0	Flavonoid
Daidzein (**104**)	8.70	C_15_H_10_O_4_	−0.31	254.0578	mzCloud	93.2	Flavonoid
Quercetin-3β-d-glucoside (**105**)	5.75	C_21_H_20_O_12_	0.62	464.0958	mzCloud	90.2	Flavonoid
(1*S*,4a*S*,6*S*,7a*S*)-4-[(β-d-Glucopyranosyloxy)methyl]-6-hydroxy-7a-methyl-7-methylene-1,4a,5,6,7,7a-hexahydrocyclopenta[c]pyran-1-yl 3-methylbutanoate (**106**)	7.79	C_22_H_34_O_10_	−0.14	458.2151	ChemSpider	55.6	Heterocyclic
1-Caffeoyl-β-d-glucose (**107**)	5.43	C_15_H_18_O_9_	0.95	342.0954	ChemSpider	73.3	Phenolic
3-[3-(β-d-Glucopyranosyloxy)-2-hydroxyphenyl]propanoic acid (**108**)	4.34	C_15_H_20_O_9_	0.62	344.1109	mzCloud	80.8	Phenolic
3-Hydroxy-3-(3-hydroxyphenyl)propanoic acid-*O*-sulphate (**109**)	3.51	C_9_H_10_O_7_S	−0.35	262.0146	ChemSpider	50.0	Phenolic
4-(3,4-Dihydroxyphenyl)-6,7-dihydroxy-2-naphthoic acid (**110**)	5.97	C_17_H_12_O_6_	0.62	312.0636	mzCloud	99.5	Phenolic
Caffeoylglycolic acid (**111**)	5.51	C_11_H_10_O_6_	−1.13	238.0475	ChemSpider	75.0	Phenolic
Esculin (**112**)	4.18	C_15_H_16_O_9_	0.23	340.0795	mzCloud	95.5	Phenolic
Salvianolic acid B (**113**)	6.62	C_36_H_30_O_16_	1.80	718.1547	mzCloud	90.5	Phenolic
2-[(2*S*,4a*R*,8a*S*)-2-Hydroxy-4a-methyl-8-methylenedecahydro-2-naphthalenyl]acrylic acid (**114**)	9.99	C_15_H_22_O_3_	−0.20	250.1568	mzCloud	86.4	Polycyclic
Dendronobiloside B (**115**)	7.31	C_21_H_38_O_8_	1.47	418.2573	ChemSpider	59.1	Terpenoid

Note: Identification of underivatized steroidal compounds via tandem mass spectrometry is impossible and any putative identity could be misleading due to their stable 4-ring skeleton and diverse stereoisomerism [22]. Thus, all matched steroidal compounds were masked and only provided with their detected molecular mass and formula. * Matching score: MS/MS Score (MzCloud)/FISh Score (ChemSpider).
